# Experiences and behaviours of patients with asthma requesting prescriptions from primary care during medication shortages linked to the COVID-19 lockdown: insights from a qualitative analysis of a UK asthma online community

**DOI:** 10.3399/BJGPO.2021.0222

**Published:** 2022-09-07

**Authors:** Nadya L Ow, Sara Sadek Attalla, Gwyneth Davies, Chris J Griffiths, Anna De Simoni

**Affiliations:** 1 Wolfson Institute of Population Health, Asthma UK Centre for Applied Research, (AUKCAR), Queen Mary University of London, London, UK; 2 Population Data Science, Asthma UK Centre for Applied Research, (AUKCAR), Swansea University Medical School, Swansea, UK

**Keywords:** asthma, coronavirus, pandemics, COVID-19, shortage, inhalers, online community, qualitative research

## Abstract

**Background:**

Inhaler shortages were reported in the UK following declaration of the COVID-19 pandemic, prompting advice against stockpiling.

**Aim:**

To understand experiences and behaviours of patients with asthma requesting prescriptions from primary care during asthma medication shortages.

**Design & setting:**

UK asthma online community, between March and December 2020.

**Method:**

Thematic analysis of posts identified using search terms ‘shortage’, ‘out of stock’, ‘prescribe’, and ‘prescription’.

**Results:**

Sixty-seven participants were identified (48 adults, two children, 17 unstated age). Factors leading to increased requests included the following: stockpiling; early ordering; realising inhalers were out of date; and doctors prescribing multiple medication items. Patients’ anxieties that could lead to stockpiling included the following: fear of asthma attacks leading to admission and acquiring COVID-19 in hospital; lack of dose counters on some inhalers; and believing a lower amount of drug is delivered in the last actuations. Strategies adopted in relation to shortages or changes in treatment owing to out-of-stock medications included the following: starting stockpiling; ordering prescriptions early; contacting medical professionals for advice or alternative prescriptions; getting ‘emergency prescriptions’; ordering online or privately; seeking medications in different pharmacies; contacting drug manufacturers; and keeping track of number of doses left in canisters. No evidence was found of anxiety-triggered asthma symptoms that required medications due to fear of COVID-19. Participants seemed to disregard advice against stockpiling.

**Conclusion:**

Better preparation is a key lesson from the COVID-19 pandemic. Clinicians, the pharmaceutical industry, and policymakers should use insights from this work to plan how to better manage medication shortages in future emergency situations.

## How this fits in

In the UK, asthma medication shortages during the first phase of the COVID-19 pandemic were followed by public messaging to stop stockpiling. However, patients' behaviour when requesting prescriptions from primary care at the time has not been reported. Anxiety, a known exacerbating factor for asthma, could itself have triggered asthma attacks, leading to legitimate increases in prescription requests. Patient factors contributing to out-of-stock medications have been identified, in particular anxiety of getting 'enough medications' to avoid exacerbations, for fear of catching COVID-19 in healthcare settings. Direct evidence was not found of asthma attacks triggered by anxiety. Difficulties estimating how much drug remains in an inhaler may have further fuelled stockpiling. Public messages about stopping stockpiling did not seem effective in changing prescription request behaviour, with participants finding different ways to obtain asthma medications, despite awareness of causing further shortages. These results require healthcare professionals, drug manufacturers, and policymakers to prepare better to address inhaler concerns and inform actions in future pandemics or situations where drug supply fails.

## Introduction

**Figure 1.**
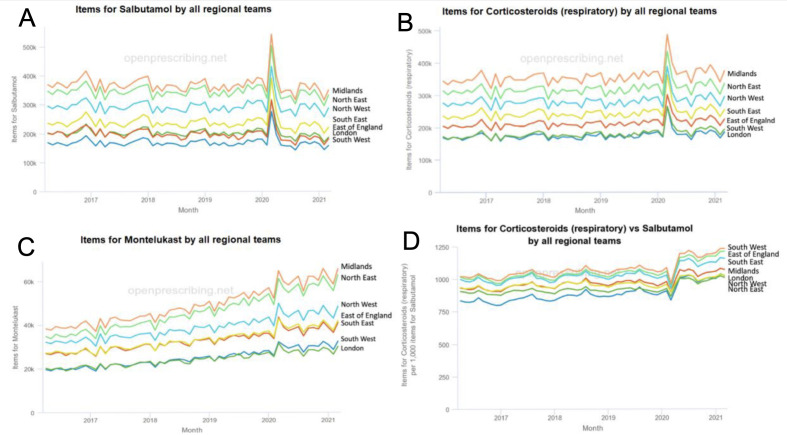
Prescription data. Panel A: bronchodilator;^6^ panel B: respiratory corticosteroids;^30^ panel C: montelukast;^31^ panel D: ratio of corticosteroids (respiratory) per each 1000 reliever prescriptions (bronchodilator), illustrating a shift towards a higher volume of corticosteroid compared with salbutamol prescriptions after the COVID-19 lockdown^32^

Over 5.4 million people with asthma in the UK are currently receiving treatment. The COVID-19 pandemic was declared by the World Health Organization (WHO) on 12 March 2020 and was associated with an unprecedented demand for steroid inhalers.^1–3^ The British Thoracic Society reported that demand for inhalers jumped by 400%, leading to shortages in the UK.^4^ Community prescription items for all medications increased by 14% in England in March, compared with February 2020.^5^ This included a 64% surge in salbutamol inhaler prescriptions (Figure 1)^6^ and 56% jump in beclomethasone inhalers within primary care.^7^ In Wales, primary care prescriptions spiked 121% and 133% for inhaled corticosteroid (ICS) and oral corticosteroids, respectively, in the week leading up to the first UK lockdown.^8^ Prescription requests for preventer ICS and reliever inhalers, as well as montelukast, reached all-time highest quantities in March 2020, after the WHO declaration and during the start of the UK’s first COVID-19 lockdown (see Figure 1).

Studying the behaviour of patients with a long-term respiratory condition, such as asthma, where anxiety is a known exacerbating factor, is of value for both the public and across primary and secondary care. Various reasons could underpin the decision to request more prescriptions, such as an increase in adherence to asthma treatment in previously poorly compliant patients. Anxiety could itself have triggered asthma attacks, leading to legitimate increases in prescription requests. However, factors driving demand of asthma medications and their relation to asthma morbidity and suspected or confirmed COVID-19 were mainly put down to stockpiling.^4^

Having a diagnosis of active asthma was associated with nearly double the odds of being diagnosed with suspected COVID-19.^8^ There is some evidence that adherence to asthma inhalers improved during the pandemic.^9^ Indeed, according to primary care data, as well as a drop in asthma exacerbations for both adults^10^ and children,^11^ there has been a significant reduction in primary and secondary care attendance for asthma exacerbations and a reduction of 36% in emergency admissions for asthma in Scotland and Wales.^8^ Patients with asthma who were diagnosed with COVID-19 reported increased inhaler use and worse asthma management, without a difference in other characteristics such as sex, ethnic group, or household income.^12^

Indeed, patients with moderate-to-severe asthma are at higher risk of suffering from complications of COVID-19,^13^ and in-hospital death.^14^

Research is needed to shed light on patients’ behaviour and factors that contributed to out-of-stock situations, to understand what led to asthma treatment shortages, how out-of-stock asthma medication was experienced by patients, why patients started stockpiling, and any consequences of not being able to access asthma medications during the pandemic. Online health communities represent views of people communicating freely with each other without time, length, or behavioural constraints that might not be captured by traditional research studies, with great potential for informing health care and policies.^15–17^ This study aimed to explore the experiences of patients with asthma with respect to COVID-19 lockdown treatment shortages, through the analysis of posts in an online community during the period March–December 2020. The objectives were to understand whether the shortage or asthma medication was discussed, reasons for requesting prescriptions, and patient perceptions and behaviours during medication shortages.

## Method

A thematic analysis of patients' posts was conducted on the online community of Asthma UK, the UK’s leading asthma research charity, as previously described.^18^ The online community comprises 18 203 people and >22 000 posts, primarily people with asthma and their carers.

Figure 1 was created by selecting relevant data from OpenPrescribing, a publicly available and free prescription cost analysis dataset, which generates graphs of time trends in prescribing across England.^19,20^

### Ethical issues

In order to protect the identity and intellectual property of participants, direct quotes have not been used, despite this being normal practice in qualitative research. Summative descriptions of quotes have been used instead throughout the article, as previously described.^16^ Description of quotes was performed by NLO. ADS checked descriptions against original quotes and the text was agreed and finalised through discussions.

### Identification of study participants

Google search engine was used to look for relevant posts within the Asthma UK community platform between March 2020 and December 2020, using the string site: https://healthunlocked.com/asthmalunguk-asthma KEYWORD/S. Usernames that were hidden by community participants were reported as ‘hidden usernames’. Different keywords were tested, with ‘shortage’, ‘out of stock’, and ‘prescribe OR prescription’ yielding relevant posts. The threads of discussions for each selected post were analysed in detail. Posts located chronologically before or after the selected posts were added to the analysis, provided they were relevant to the research questions. Relevant posts were copied and pasted into an Excel database for later analysis. Username, names, or pseudonyms; sex; age; asthma treatment; whether participant was a person with asthma or a patient discussed by third party; and third-party relation with patient (for example, parent) were retrieved where available within the posts, as previously described.^18^

### Analysis

NLO and SSA analysed the posts using thematic analysis,^21^ using a data-driven approach. More details on the application of thematic analysis on this source of data are provided in a previous study.^18^ NLO coded all posts separately, according to the type of asthma medications. Themes and strategies emerging from posts were similar across asthma medications (preventer versus relievers), as well as for shortage and out-of-stock prescriptions, and therefore results were subsequently analysed and presented together.

Coding was performed independently by SS on 20% of posts, and discussed until agreement was reached.

Throughout the article the term ‘anxiety’ is used in relation to the concerns expressed by participants about drug shortages, rather than a diagnostic term.

## Results

The keywords search identified 271 posts, 136 of which (50.2%) were relevant to the research questions and were vincluded in the analysis.

### Participants

A total of 67 participants were identified (Table 1). The majority of users did not state their exact age. Among participants whose sex was revealed within posts, females were about 10 times as numerous as males. No participant mentioned their ethnic background. The average number of posts per participant was 1.9. Some participants contributed with more than one post; for example, one wrote nine, one wrote six, and six participants wrote five posts each.

**Table 1.** Characteristics of the Asthma UK community participants as ascertained from their posts, and treatments mentioned by participants

Sample characteristics	*n*
**Number of unique usernames, names, or pseudonyms** (Hidden usernames excluded)*	**67**
Adults with asthma	48
Children with asthma talked about by third party	2
Unspecified age	17
**Sex of participants**	
Female	34
Male	3
Usernames with unspecified sex	30
**Number of posts**	***n***
**Total number of posts**	**136**
Posts by hidden usernames	11
**Treatment (number of posts stating type of treatment**)	
Preventer inhalers	34
Reliever inhalers	37
Combined inhalers	33
Montelukast	18
Injections	4
Antihistamines	7
Nebuliser or nebules	27
Nasal spray	7
Prednisolone tablets	4
Magnesium (intravenous)	1
Not stated	13

*This number does not include participants from the ‘hidden username’ group, which could not be accounted for.

### Themes

Themes and sub-themes are shown in Table 2.

**Table 2.** Themes divided into factors, concerns, and strategies used by members of an online asthma community

Coding	Sub-themes
Factors leading to increased prescription requests	Patients stockpiling Doctors prescribing more Realising inhalers were out of date Requesting new treatment for fear of being admitted to hospital during the pandemic Belief that asthma treatment could be used to treat COVID-19 symptoms Original treatment being out of stock Request of multiple prescriptions to avoid prescription charge Beliefs about reduced dose towards end of use of inhalers Buying extra inhalers owing to difficulty with tracking remaining doses without counters
Concerns towards lack of and/or change in treatment	Deterioration of medical condition Healthcare professionals not being aware of the shortage Pharmacies not having patients’ interests at heart
Patients’ and healthcare professionals’ strategies to deal with lack of or change in treatment	Change of treatment Contacting healthcare providers, out-of-hours health care, pharmacies, online pharmacies, and private healthcare services Individually driven check of asthma medication shortages Keeping track of doses when canisters have no counter

Results were grouped according to the following four main themes:

1. Availability of asthma medications (this section is a factual description of the shortages experienced by users, and has been omitted from Table 2)
2. Factors leading to increased prescription requests
3. Concerns about lack of and/or change in treatment
4. Patients’ and healthcare professionals' strategies to deal with lack of and/or change in treatment.

#### Availability of asthma medications

Clenil Modulite inhalers were reported to be out of stock in several posts between June and October 2020, whereas other preventer inhalers such as Qvar and Kelhale were available. Some participants could get Alvesco prescriptions, while others found them to be out of stock. Reliever inhalers such as Ventolin were mostly reported to be available; however, some reported having to wait longer (up to a few weeks) to collect their prescriptions.

Combined inhalers such as Sirdupla and Symbicort were available, but Fostair was mentioned by some as out of stock or needing longer for prescription to be dispensed.

Montelukast was reported to be out of stock, or when available, needing to be requested at least a week in advance.

Nebulisers, albeit unspecified as to whether this referred to nebuliser machines and/or nebuliser vials, were also in shortage.

Prednisolone tablets, antihistamines, nasal sprays, and antibiotics were reported as available.

#### Factors leading to increased prescription requests

##### Patients stockpiling and doctors prescribing more

Stockpiling or observed stockpiling of asthma medications (that is, participants reporting other users writing about getting multiple prescriptions) was mentioned for preventer inhalers, combined inhalers, and montelukast, despite awareness of it causing further shortages.

A mother of an adult with asthma was able to receive montelukast, albeit after 1 week of waiting. She hypothesised that others were requesting prescriptions before they needed them and stockpiling during the first lockdown. She stated that she too would start ordering prescriptions early, despite acknowledging that this would add to the problem. (Female, adult, exact age not stated, mother of adult with asthma, participant N.8, April 2020)

There was evidence that healthcare professionals could in part contribute to the shortage of medications through increasing the number of items on each prescription.

An adult participant likened the stockpiling of medicine to stockpiling of toilet roll, and described that medical professionals were prescribing patients three or four inhalers before they were needed, thus leading to the shortage after the first lockdown was announced. (Sex not stated, adult, exact age not stated, participant N.4, May 2020)

##### Realising inhalers were out of date

Another reason for requesting prescriptions was noticing that current prescription medications were beyond their expiry date.

During the month leading up to the first lockdown, a participant realised their reliever inhalers [one to be used and one spare] were out of date. They felt that it would be wise to be prepared during the pandemic and hence requested a repeat prescription for these from their GP. (Sex not stated, age not stated, participant N.53, March 2020)

Some participants expressed the need to obtain additional prescriptions in case of an asthma attack to avoid hospital admissions, especially owing to the fear of being admitted to a COVID-19 ward.

A participant explained how during the start of the pandemic she was in hospital needing nebulisers, and because she was coughing, she was admitted to a COVID ward. She stated she would prefer getting a home nebuliser and taking her specialist-prescribed emergency steroids to avoid being admitted to the hospital. (Female, adult, participant N.33, October 2020)

##### Belief that asthma treatment could be used to treat COVID-19 symptoms

Some participants raised queries about whether asthma treatments were effective in the management of COVID-19 symptoms, which could contribute further to stockpiling.

A participant responded to queries from users under the impression that asthma medications were being used to treat COVID. She responded that inhalers should be taken regularly as prescribed to control asthma, but that they are not a treatment for COVID symptoms. (Female, adult, exact age not stated, participant N.66, June 2020)

##### Changes in treatment owing to out-of-stock medications

Participants reported changes in preventer inhaler brands or switching from branded to generic inhalers, owing to shortages.

A participant was switched to 200 mg Clenil Modulite 1 puff twice daily from Clenil 100 mg 2x puffs twice [doubling the dose but halving the number of puffs] because of the original treatment being out of stock. (Sex not stated, age not stated, participant N.45, June 2020)

##### Beliefs about reduced doses towards end of inhaler canister

Some participants described the belief that reduced doses were released in each actuation towards the end of each inhaler leading to an increased request for prescriptions.

A participant describes taking extra doses of her combined inhaler whenever there are below 20 doses left on her inhaler canister’s counter. She remarked that the last few inhaler puffs seem to deliver a reduced dose, based on the sound it would make during each puff. This meant that she had to order more combined inhalers, not just because of giving herself the additional doses, but also as she preferred to have a spare towards the end of her inhaler’s doses. (Female, exact age not stated, participant N.63, June 2020)

##### Difficulty with tracking remaining doses without counters

Another reason that could lead to increased requesting of spare reliever inhalers was that the lack of consistent pattern of use made it difficult to keep track of the remaining number of doses left in the canister.

An adult with asthma agreed with other users that it was strange for reliever inhalers not to have counters, as this meant she would need to track them by herself. She noted that even after the doses in her reliever inhaler were used up, there would still be propellant coming out for numerous puffs, so patients would be getting propellant-only doses unless they recorded the number of puffs they have used. (Female, adult, exact age not stated, participant N.44, March 2020)

### Concerns about lack of and/or change in treatment owing to out-of-stock medications

#### Risk of deterioration of medical condition

Participants showed anxieties about the potential worsening of their asthma owing to their treatment being not available, especially if this would lead to a hospital admission and the risk of getting COVID-19 there. This made them even more anxious about treatment shortages and cautious about changing medication.

A mother speaking on behalf of her 6-year-old son worried about the shortage of his preventer inhaler, which was out of stock everywhere. She feared that without it, his condition would get worse, and he would require a hospital admission, which she thought should be avoided especially during the pandemic. (Male, child, aged 6 years, participant N.40, October 2020)

#### Healthcare professionals being unaware of the shortage

Some participants remarked that their healthcare professionals were not aware of the shortage and thus kept prescribing treatments that were not available.

A participant stated that they were facing preventer inhaler shortages, and that their pharmacist had suggested alternatives. They commented about a fellow participant’s asthma nurse who continued prescribing the preventer inhaler wondering if the nurse had known it was in shortage. (Sex not specified, adult, exact age not stated, participant N.64, October 2020)

#### Pharmacies not having patients’ interests at heart

Some patients worried about pharmacies possibly withholding medications depending on the asthma medication brand profitability.

An adult patient reported being unable to acquire a branded reliever inhaler at a named pharmacy chain until they cited having contacted the manufacturers, who reported no shortage, before being able to receive the inhalers. They noted this happened two times in a row and posited that pharmacies could be motivated by profit from certain brands. (Sex not specified, adult, exact age not stated, participant N.50, March 2020)

### Patients’ and healthcare professionals’ strategies to deal with lack of or change of treatment owing to medication shortages

An interesting range of suggestions were put forth by users of the online community to support one another in the face of shortages or changes in treatment.

#### Change of treatment

Participants who were unable to obtain their original prescriptions would receive or seek different treatments. This could be a change from branded to generic treatments, a change in dose of the same treatments, or a change in treatment regimens entirely.

A participant described how their GP surgery explained that because of a global shortage they were only able to obtain an inhaler double the dose of the initial one. The GP surgery prescribed the higher dose inhaler but advised them to half the number of puffs taken. (Sex not specified, age not stated, participant N.45, June 2020)

#### Contacting healthcare providers

Participants who experienced treatment shortages also often shared about contacting healthcare professionals through pharmacies, hotlines, or via arranging consultations to obtain medical advice.

A father writing on behalf of his child with asthma was unable to obtain her usual beclomethasone dipropionate inhaler [Clenil] and approached healthcare professionals for a suitable alternative. They were prescribed an inhaler with the same active ingredient [Qvar], but in superfine particles [and hence was twice as potent]. (Female, child, aged 4 years, participant N.5, October 2020)

Some participants were able to get out-of-stock medication through bigger pharmacy chains.

One participant described obtaining their reliever inhaler through a named pharmacy chain, rather than smaller individually owned pharmacies. (Sex not specified, exact age not stated, participant N.61, October 2020)

A participant suggested taking a previous preventer inhaler prescription to any pharmacy in an emergency. She also suggested calling 111 to receive an out-of-hours prescription, which can be dispensed at any pharmacy. As for reliever inhalers, she advised that it was possible to bring an empty reliever inhaler, with or without the previous prescription slip, to a pharmacy as evidence to get an emergency prescription without verifying with GPs. (Female, adult, exact age not stated, participant N.24, month unspecified, 2020)

Another participant also advised fellow users to request inhalers via local pharmacies on weekdays, while on weekends, she advised them to call 111 or go to the A&E department to obtain inhalers that have run out without a new regular prescription. (Female, adult, exact age not stated, participant N.51, month unspecified, 2020)

#### Individually driven check of asthma medication shortages

Participants also suggested checking with manufacturers, as well as with distributors about availability of treatments.

A participant described that they only found official notice of a named preventer inhaler shortage in September 2018. They described contacting manufacturers in a previous episode in which combined inhalers were in short supply, to which the manufacturers informed them that there was no shortage at the source, merely an issue with inhaler distribution. (Sex not specified, adult, exact age not stated, participant N.50, October 2020)

#### Keeping track of doses when canisters have no counter

Participants also discussed methods to keep track of inhaler doses for canisters with no counters.

One participant suggested checking remaining doses in pressurised canister inhalers by submerging the canister in water, but that this was not applicable for dry powder inhalers. Full canisters would sink while empty ones would float. Therefore, canisters with doses left in them would not fully sink to the bottom. (Male, adult, aged 75 years, participant N.62, month unspecified, 2020)

An adult participant advised fellow users to record the number of doses used per each inhaler without a counter on their peak flow charts. (Female, adult, aged 71 years, participant N.32, month unspecified, 2020)

## Discussion

### Summary

This novel study has shed light on the experience and behaviours of patients with asthma requesting prescriptions during the lockdowns and an initial out-of-stock inhaler episode, as well as their concerns and strategies to cope with medication shortages, and alternative drug regimens started as results of out-of-stock prescriptions. The COVID-19 pandemic led to disruption to asthma medication supplies and changes to patient medication ordering. Patients with asthma suffered anxiety as a result of asthma medication shortages, sharing their experiences online.

Participants described shortages of certain preventer and combined inhalers, montelukast, and nebuliser vials, while also reporting longer waiting times for reliever treatment. Anxiety about the shortage of inhalers could well have triggered asthma symptoms and hence additional legitimate prescription requests, although direct mention of this was not found. Participants ordered prescriptions earlier than needed, or ordered these extra prescriptions on top of their regular ones, despite awareness that extra prescriptions could further fuel the shortage. Not having a dose counter on inhalers, and the beliefs that lower doses of medications are delivered in actuations towards the end of their inhalers, could also have led to an increase in prescription requests and potential overdosing of medication. Asthma medications were valued and treasured. These data triangulate with, confirm, and extend available evidence and are relevant.^1–9,10,10^

### Strengths and limitations

A strength of this work lies in the spontaneous nature of the data provided by online communities. Such data are less likely to be affected by self-presentation, reactivity, and recollection biases and by the influence of the researcher’s agenda.^15,22^

The main limitations of this approach are potential biases in the sample of participants (that is, patients with asthma who took part in an online asthma community), limited information on participants’ background characteristics, which might have affected representativeness, and the inability to ask follow-up questions to participants.

The search was limited to words ‘shortage’, ‘prescribe’ or ‘prescription’ and ‘out of stock’ and therefore might have missed posts where none of these keywords were present.

### Comparison with existing literature

The data supports that asthma prescription requests were increased, especially during the first COVID-19 lockdown.^1–3^ While the increase in prescription requests leading to medication shortages at the start of the pandemic is understandable, as there was uncertainty about what would happen to asthma care both from patients and primary care clinicians, whether the NHS would be overwhelmed, and what access patients would have to health care and/or prescriptions, the qualitative data contextualised the previously known statistics, providing factors leading to increased prescription requests.

Current evidence has shown that patients have been less likely to present for asthma exacerbations during the pandemic.^10,23^ This is in line with the present study’s findings, as it was found that patients were doing their best to avoid admissions through stockpiling their regular prescription medications, such as asthma inhalers, as well as obtaining new prescriptions. The data do not exclude that the increased ICS prescription requests during the first lockdown was triggered by increased overall adherence to preventer inhalers, rather than patients merely stockpiling for spares, although direct evidence of this was not found. Indeed, the reduction in reliever inhaler requests after the first lockdown could well be owing to better adherence to ICS and the consequent better asthma control (Figure 1, panel D). The ratio of ICS to bronchodilator across the UK regions remained higher from March 2020 coupled with reduced asthma exacerbations in primary and secondary care,^9–23,23^ which could suggest a continued change in patient behaviour and improvements in asthma self-management; that is, increased prescription request (and potentially adherence) to ICS from the first COVID-19 lockdown (Figure 1).

The perception that less drug dose is delivered towards the end of inhalers was not false, as inhaler drug dose distribution varies. The main factor depends on if it is suspension- or solution-based. Solution-based inhalers contain drug particles uniformly distributed in propellant, while suspension-based inhaler drug particle distribution depends on the density of drug particles in comparison with the density of propellant.^24^ Examples of suspension-based inhalers include Symbicort, Ventolin, and Seretide, while a solution-based inhaler is Clenil.^24^ Suspension-based emitted doses are dependent on multiple factors such as storage and shaking before use.^25^ Dose towards the end of suspension-based inhalers depends on multiple factors such as the ratio of propellant to drug,^26^ and density of drug compared with propellant. For example, one study^24^ found that when Seretide and Ventolin inhalers were not shaken before use, there was an increase in the amount of drug delivered at the start of the canister life, whereas for Symbicort inhalers, the amount of the drug delivered increased at the end of the unshaken canister life. Clinicians should familiarise themselves with drug delivery in different inhalers, discuss concerns about drug concentration in actuations and address them, also to avoid risk of overdosing.

Dose counters were also favoured by participants, as supported in other studies.^27–29^ However, dose counters are still not a component of all inhaler types. Being aware of the number of actuations left helps patients with timing when to order new inhalers, and could potentially prevent inhalers being discarded when the drug is still present, avoiding unnecessary waste, or continued when no drug remains.

### Implications for research and practice

Anxieties surrounding asthma control and hospitalisation were a factor in COVID-19 lockdown-related increased prescription requests. Anxiety itself could have caused asthma attacks, which would increase patients’ use of inhalers. In the event of future respiratory disease epidemics, encouraging patients not to stockpile could have minimal effects on inhaler demand and patient behaviour, unless patient concerns and behaviours are properly understood and addressed by their healthcare providers. Indeed, evidence was found that participants kept stockpiling despite being aware that such behaviour contributed further to drug shortages.

Ordering prescriptions from online pharmacies could potentially mask how often patients are requiring inhalers. This could be an important question to clarify with patients during their asthma reviews, as they could be using additional inhalers than they are being prescribed, masking the degree of their asthma severity and control.

## Data sharing statement

This is not applicable, as the data was not collected for this study is widely available on Internet. Asthma UK and HealthUnlocked have approved the study protocol.

## Data

This is not applicable, as the data was not collected for this study is widely available on the internet. Asthma UK and HealthUnlocked have approved the study protocol.

## References

[1] Elbeddini A (2020). Sterilization plan of the used metered dose inhalers (MDI) to avoid wastage amid COVID-19 pandemic drug shortage. J Pharm Policy Pract.

[2] Amirav I, Newhouse MT (2020). Asthma and COVID-19: in defense of evidence-based SABA. J Asthma Allergy.

[3] Hendeles L, Prabhakaran S (2020). Nationwide shortage of albuterol inhalers and off-label use in COVID-19 patients. Pediatr Allergy Immunol Pulmonol.

[4] Mahase E (2020). Covid-19: increased demand for steroid inhalers causes “distressing” shortages. BMJ.

[5] NHS Business Services Authority (2020). Prescription Cost Analysis (PCA) data.

[6] OpenPrescribing Salbutamol: BNF Code 0301011R0 [results shown: 2016 to 2021]. https://openprescribing.net/chemical/0301011R0/.

[7] OpenPrescribing Beclomethasone: BNF Code 0302000C0. https://openprescribing.net/chemical/0302000C0/.

[8] Hull SA, Williams C, Ashworth M (2020). Prevalence of suspected COVID-19 infection in patients from ethnic minority populations: a cross-sectional study in primary care. Br J Gen Pract.

[9] Kaye L, Theye B, Smeenk I (2020). Changes in medication adherence among patients with asthma and COPD during the COVID-19 pandemic. J Allergy Clin Immunol Pract.

[10] Shah SA, Quint JK, Nwaru BI, Sheikh A (2021). Impact of COVID-19 national lockdown on asthma exacerbations: interrupted time-series analysis of English primary care data. Thorax.

[11] Gupta A, Bush A, Nagakumar P (2020). Asthma in children during the COVID-19 pandemic: lessons from lockdown and future directions for management. Lancet Respir Med.

[12] Philip KE, Buttery SC, Williams P (2022). Impact of COVID-19 on people with asthma: a mixed methods analysis from A UK wide survey. BMJ Open Respir Res.

[13] Lee SC, Son KJ, Han CH (2020). Impact of comorbid asthma on severity of coronavirus disease (COVID-19). Sci Rep.

[14] Williamson E, Walker AJ, Bhaskaran K (2020). OpenSAFELY: factors associated with COVID-19-related hospital death in the linked electronic health records of 17 million adult NHS patients. Nature.

[15] De Simoni A, Shanks A, Mant J, Skelton JR (2014). Making sense of patients’ internet forums: a systematic method using discourse analysis. Br J Gen Pract.

[16] Balasooriya-Smeekens C, Bateman A, Mant J, De Simoni A (2016). Barriers and facilitators to staying in work after stroke: insight from an online forum. BMJ Open.

[17] De Simoni A, Shanks A, Balasooriya-Smeekens C, Mant J (2016). Stroke survivors and their families receive information and support on an individual basis from an online forum: descriptive analysis of a population of 2348 patients and qualitative study of a sample of participants. BMJ Open.

[18] De Simoni A, Horne R, Fleming L (2017). What do adolescents with asthma really think about adherence to inhalers? Insights from a qualitative analysis of a UK online forum. BMJ Open.

[19] Curtis HJ, Goldacre B (2018). OpenPrescribing: normalised data and software tool to research trends in English NHS primary care prescribing 1998–2016. BMJ Open.

[20] Walker AJ, Curtis HJ, Croker R (2019). Measuring the impact of an open web-based prescribing data analysis service on clinical practice: cohort study on NHS England data. J Med Internet Res.

[21] Braun V, Clarke V (2006). Using thematic analysis in psychology. Qual Res Psychol.

[22] Jamison J, Sutton S, Mant J, De Simoni A (2018). Online stroke forum as source of data for qualitative research: insights from a comparison with patients’ interviews. BMJ Open.

[23] Davies GA, Alsallakh MA, Sivakumaran S (2021). Impact of COVID-19 lockdown on emergency asthma admissions and deaths: national interrupted time series analyses for Scotland and Wales. Thorax.

[24] Chierici V, Cavalieri L, Piraino A (2020). Consequences of not-shaking and shake-fire delays on the emitted dose of some commercial solution and suspension pressurized metered dose inhalers. Expert Opin Drug Deliv.

[25] Everard ML, Devadason SG, Summers QA, Le Souëf PN (1995). Factors affecting total and “respirable” dose delivered by a salbutamol metered dose inhaler. Thorax.

[26] Michael Y, Snowden MJ, Chowdhry BZ (2001). Characterisation of the aggregation behaviour in a salmeterol and fluticasone propionate inhalation aerosol system. Int J Pharm.

[27] Crompton GK (2004). How to achieve good compliance with inhaled asthma therapy. Respir Med.

[28] Conner JB, Buck PO (2013). Improving asthma management: the case for mandatory inclusion of dose counters on all rescue bronchodilators. J Asthma.

[29] Fish L, Lung CL, Antileukotriene Working Group (2001). Adherence to asthma therapy. Ann Allergy Asthma Immunol.

[30] OpenPrescribing Total prescribing for corticosteroids (respiratory) across all regional teams in NHS England [results shown: 2016 to 2021]. https://openprescribing.net/analyse/#org=regional_team&numIds=3.2&denom=nothing&selectedTab=chart.

[31] OpenPrescribing Total prescribing for montelukast across all regional teams in NHS England [results shown: 2016 to 2021]. https://openprescribing.net/analyse/#org=regional_team&numIds=0303020G0AA&denom=nothing&selectedTab=chart.

[32] OpenPrescribing Total prescribing for corticosteroids (respiratory) per each 1000 reliever prescriptions (bronchodilator) across all regional teams in NHS England [Results shown: 2016 to 2021]. https://openprescribing.net/analyse/#org=regional_team&numIds=3.2&denomIds=3.1&selectedTab=chart.

